# Acute Myocardial Infarction Due to Metastatic Melanoma Tumor Embolization

**DOI:** 10.1016/j.jaccas.2024.102793

**Published:** 2024-12-04

**Authors:** Ahmed Saleh, Syed Hyder, Usman A. Hasnie, Edward Chung, Paul Benson, Darryl Prime, Carrie Lenneman, Samuel McElwee

**Affiliations:** Division of Cardiology, University of Alabama at Birmingham, Birmingham, Alabama, USA

**Keywords:** coronary interventions, immune checkpoint inhibitor–induced colitis, intravascular ultrasound, melanoma, myocardial infarction

## Abstract

Embolic myocardial infarction (MI) due to malignancy is a rare clinical entity defined by embolization of cancerous tissue to the coronary arteries. We present the case of a 49-year-old man presenting with an anterolateral MI in the setting of metastatic melanoma tumor embolization.

## Graphical Abstract

**Figure:**
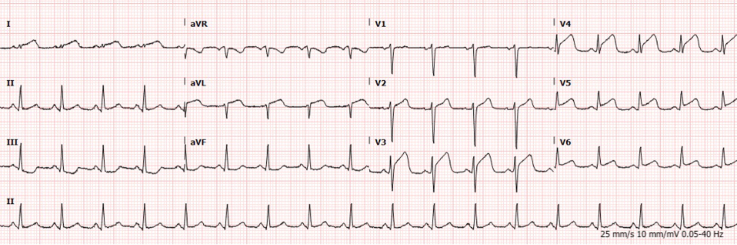

## History of Presentation

A 49-year-old man with oligometastatic melanoma presented to the emergency department with 1 hour of crushing left-side chest pain radiating to the neck as well as dyspnea. He was started on immunotherapy after a diagnosis of oligometastatic melanoma 4 months earlier and had completed his fourth cycle 1 month before the onset of his chest pain. Two weeks before presentation, he was also diagnosed with biopsy-confirmed severe immune checkpoint inhibitor–related colitis secondary to ipilimumab and nivolumab and was being treated with high-dose steroids for 5 days followed by a 2-week course of prednisone taper. Vital signs in the emergency department revealed hypotension with a blood pressure of 93/56 mm Hg and tachycardia with a heart rate of 113 beats/min. Cardiac physical exam was notable for a regular rhythm and no appreciable elevation of jugular venous pulsation.

**Learning Objective**

• To identify a rare etiology of acute coronary syndrome in patients with metastatic melanoma.

## Past Medical History

The patient had oligometastatic melanoma treated with ipilimumab and nivolumab. He had a history of diabetes with an HgA_1c_ of 6.7%, but no history of tobacco use, hypertension, hyperlipidemia, or other cardiovascular disease risk factors.

## Differential Diagnosis

The differential diagnosis included coronary vasospasm, myocarditis, eccentric plaque rupture, and coronary embolism.

## Investigations

**Figure 1:**
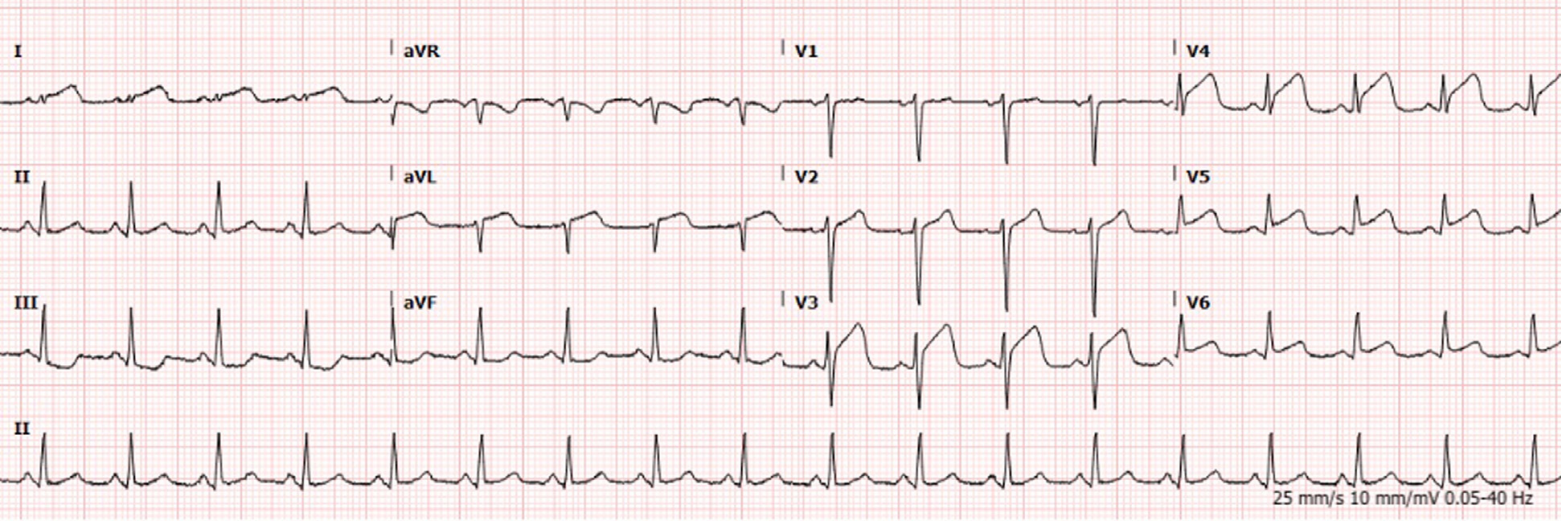
Preprocedural ECG Preprocedural electrocardiogram showing ST-segment elevations in the anterior lateral leads with reciprocal changes.

Electrocardiography revealed 2-mm ST-segment elevations in the anterolateral leads, accompanied by reciprocal ST-segment depression in the inferior leads (Figure 1). His initial laboratory evaluation was significant for an elevated high-sensitivity troponin level of 134 ng/L (reference range: 3-20 ng/L) that increased to 10,029 ng/L within 3 hours. Creatinine kinase level was 900 U/L. Laboratory assessment of atherosclerotic cardiovascular risk revealed normal cholesterol (LDL 40 mg/dL) and HgA_1c_ of 6.5%. Echocardiography revealed newly reduced ejection fraction with apical hypokinesis.

## Management

**Figure 2:**
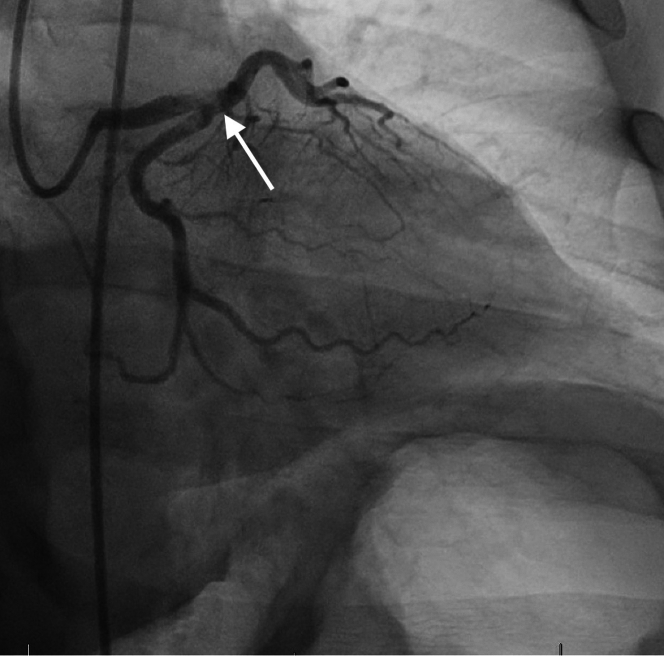
Coronary Angiography of Prior to Intervention Coronary angiography showing intraluminal filling defect of the distal left main, proximal left anterior descending, and ostial to proximal left circumflex arteries.

**Figure 3:**
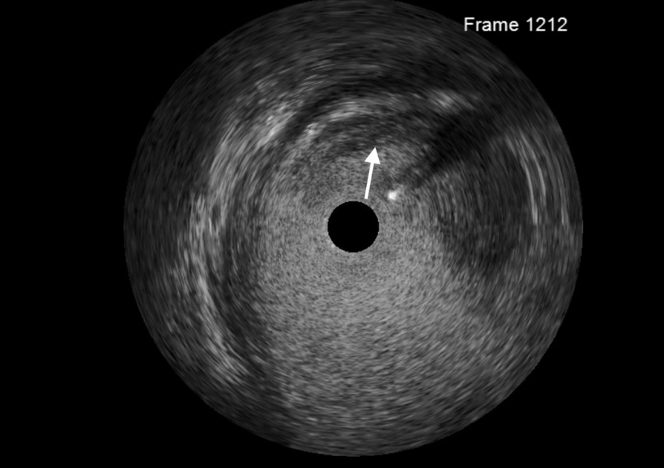
Intravascular Ultrasound of Distal Left Main Coronary Artery Intravascular ultrasound of the distal left main artery showing a lesion with mixed echoic and hypoechoic characteristics representing thrombus.

**Figure 4:**
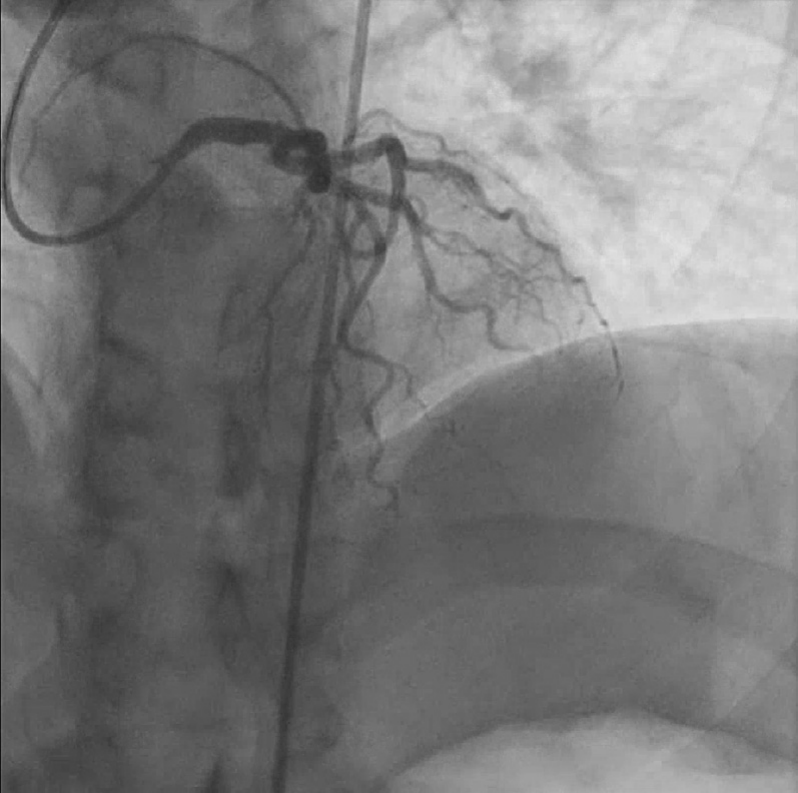
Coronary Angiography Following Intervention Coronary angiography showing restored flow to the distal left anterior descending artery after balloon angioplasty.

**Figure 5:**
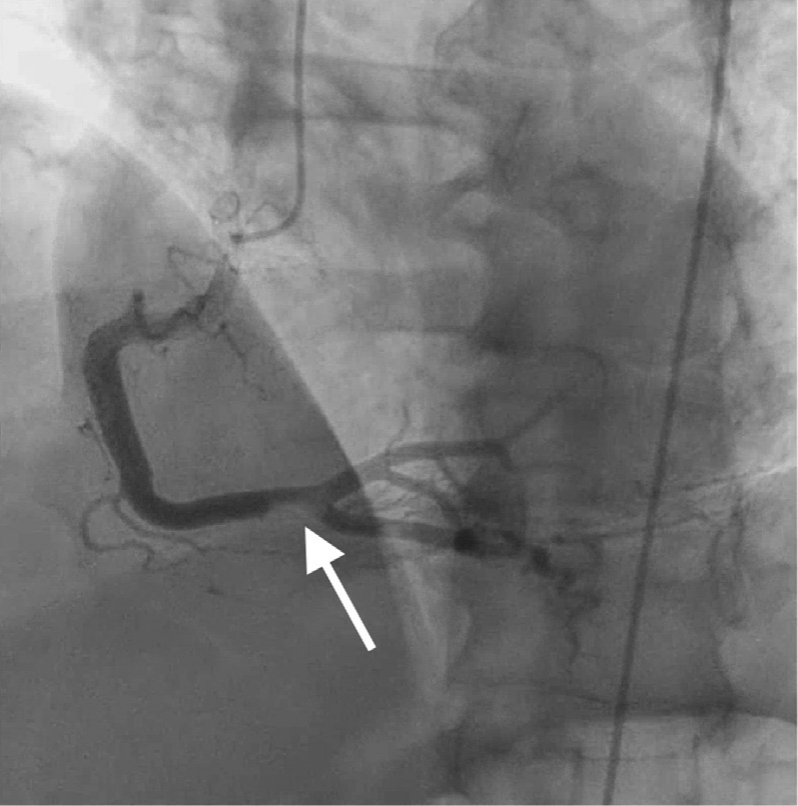
Coronary Angiography of Right Coronary Artery Coronary angiography showing intraluminal filling defect of the distal right coronary artery.

The patient was taken for emergency left heart catheterization after initiation of 325 mg aspirin, 190 mg ticagrelor, and full-dose heparin. Coronary angiography confirmed filling defects of the distal left main (LM), proximal left anterior descending (LAD), and ostial and proximal left circumflex (LCX) coronary arteries. Thrombus was observed within the distal LM extending to the ostial LAD and ostial LCX (Figure 2, Video 1). Intravascular ultrasound (IVUS) was performed, which also showed extensive thrombosis without evidence of native coronary artery disease throughout the anterior circulation (Figure 3, Videos 1 and 2). PCI was considered but ultimately deferred owing to unfavorable caliber of the distal LAD, concern for bleeding risk on dual antiplatelet therapy in the setting of known gastrointestinal bleeding with immune checkpoint inhibitor–related colitis, and risk of in-stent restenosis. Multiple balloon dilatations were performed along the length of the distal LM and proximal LAD. TIMI flow grade II and the disappearance of the thrombotic lesion were then visualized (Figure 4, Video 3). Angiography of the right coronary artery (RCA) revealed thrombus within the distal portion of the vessel; however, TIMI flow grade III was present, so intervention was not pursued (Figure 5). Postprocedure transesophageal echocardiography was performed, which did not show evidence of shunting, valvular vegetation, apical thrombus, or atrial thrombus.

**Figure 6:**
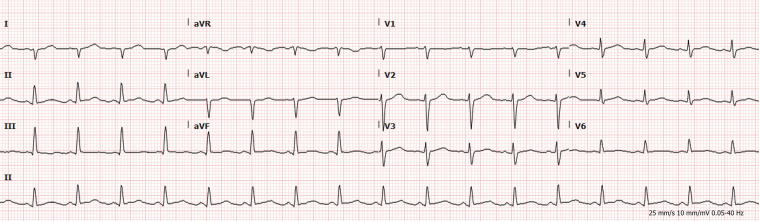
Postprocedural ECG Postprocedural electrocardiogram showing resolution of ST-segment elevations.

**Figure 7:**
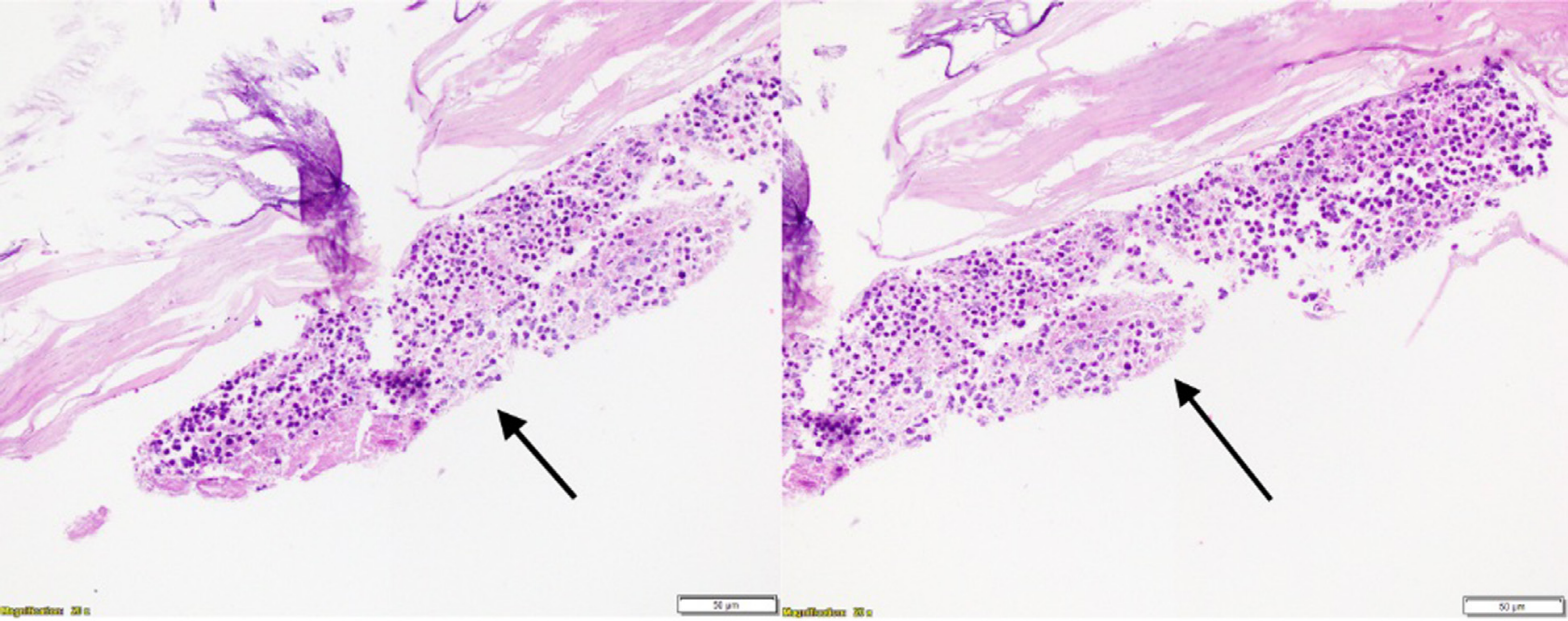
Intraluminal Thrombus High-power hematoxylin and eosin staining of left main coronary artery with intraluminal thrombus (arrows) with embedded melanocytes and coronary intima without metastatic involvement. The coronary artery endothelium was compromised in preparation of the slide, giving the false appearance of a stalk.

**Figure 8:**
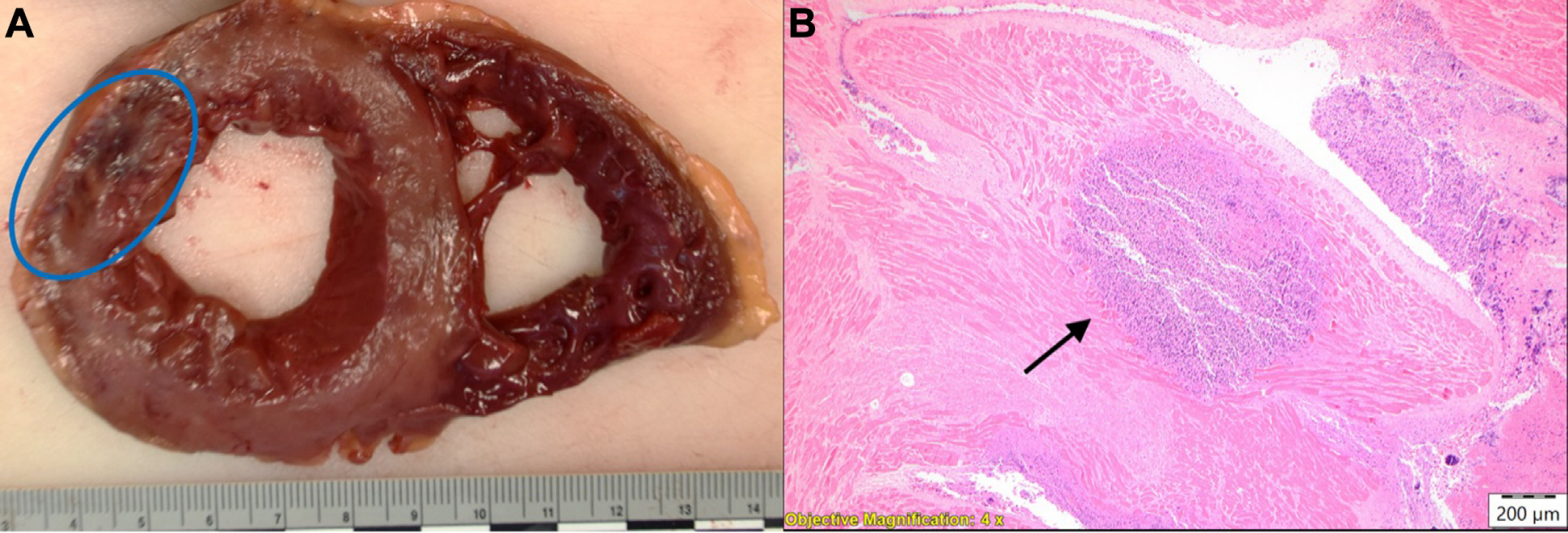
Myocardial Necrosis and Evidence of Cardiac Metastasis (A) Gross dissection of the left ventricle showed an area of myocardial necrosis (blue circle). (B) Dense large nests of tumor cells infiltrating the myocardium with necrosis (arrow), suggesting cardiac metastasis.

On return from the catheterization laboratory, the patient was continued on 81 mg aspirin and 90 mg ticagrelor twice daily. Before discharge, he was changed to apixaban 5 mg twice daily owing to high suspicion that the etiology of his myocardial infarction (MI) was embolic based on multivessel involvement, IVUS appearance, and lack of native coronary artery disease. Postprocedural electrocardiography and echocardiography obtained the following day revealed the resolution of acute ST-segment changes and apical hypokinesis, with an ejection fraction of 50%-60% (Figure 6). He remained hospitalized for 3 days and had complete resolution of his chest pain. One month later, he was admitted to an outside hospital for sepsis and died after developing distributive shock. An autopsy revealed intraluminal clusters of melanocytes embedded within thrombus in the LM, excluding the coronary artery smooth muscle. These findings suggest tumor embolization within an evolving thrombus, leading to acute coronary syndrome (Figure 7). There was minimal calcific atherosclerosis (10%-20%) in the left and right coronary circulation. In addition, histologic staining revealed clusters of melanoma within the myocardium, including the left ventricular wall and ventricular septum within the bundle branch, indicating cardiac metastasis (Figure 8). Notably, there were no melanocytes or lymphocytic predominance on coronary histology to suggest metastasis to the coronaries or vasculitis.

## Discussion

This case highlights a rare instance of tumor embolization in the coronary arteries causing acute MI. Postmortem histology found melanocytes within thrombus in the LM, LAD, LCX, and RCA, without evidence of melanoma affecting the coronary arteries’ smooth muscle, making tumor-mediated rupture unlikely. Despite the unknown embolic source, the patient’s extensive tumor burden, histologically confirmed melanocytes, and absence of atherosclerosis on IVUS or angiography suggest that the acute coronary syndrome was likely due to tumor embolization rather than plaque rupture or erosion.

Tumor embolization leading to acute MI is a rare phenomenon. A systematic review in 2022 identified lung cancer and atrial myxoma as the most common sources of arterial tumor embolism. Acute MI was reported in 21.4% of the case reports reviewed.^1^ Among cohorts of patients with stage IV melanoma, arterial thrombosis is quite common, with one study reporting roughly 6.1% of patients having an event, and 35.7% of events being acute MI.^2^ In addition, there are several reported cases of metastatic melanoma embolizing to arterial sites, including the lower extremities and cerebral arteries, resulting in occlusion.^3^^,^^4^ In these cases, the embolic sources of the metastatic disease were thought to originate from cardiac metastasis.

Melanoma is one of the most common cancers to metastasize to the heart, with estimates of 28% to 56% of patients with metastatic melanoma having some cardiac involvement.^5^ A combined retrospective study and systematic review in 2023 revealed the most common locations for cardiac involvement to be endocardial locations, including the left ventricle, right ventricle, and right atrium.^6^ This is unlike other malignancies, which tend to involve the pericardium and epicardium. These findings may be explained by melanoma’s propensity for hematologic and lymphatic spread compared with other tumors. It may also explain a potential mechanism for arterial embolization in these patients.

Advanced melanoma with cardiac metastasis portends a short life expectancy, with an estimated 2-year survival of 59%.^6^ Although there are no existing guidelines for the management of metastatic coronary embolization, routine aspiration thrombectomy is not recommended for the treatment of acute MI with significant thrombotic burden, owing to a lack of evidence showing improvement in clinical outcomes compared with percutaneous coronary intervention alone.^7^ Long-term oral anticoagulation is generally recommended for empiric treatment of suspected coronary embolism. However, anticoagulation is unlikely to protect against recurrence if tumor growth cannot be controlled.^8^ Although antiplatelet therapy is recommended for acute MI in the setting of plaque rupture or erosion secondary to atherosclerosis, it remains unclear if these therapies are effective in the setting of coronary embolization.

## Follow-Up

After the patient’s death, his family had requested an autopsy.

## Conclusions

This case report presents a unique instance of MI caused by the tumor embolization. Though cardiac involvement of melanoma is not unusual, this is the first documented case report of an MI due to melanoma-embedded thrombi. In patients with known metastatic melanoma presenting with symptoms of acute coronary syndrome, we urge providers to consider a tumor embolism as an etiology, especially in the absence of atherosclerotic coronary artery disease.

## Funding Support and Author Disclosures

The authors have reported that they have no relationships relevant to the contents of this paper to disclose.

## Appendix

Video 1 Video of angiography of anterior circulation.

Video 2 Video of intravascular ultrasound of the anterior circulation.

Video 3 Video of angiography of anterior circulation after balloon angioplasty.
